# Phylogeography in Response to Reproductive Strategies and Ecogeographic Isolation in Ant Species on Madagascar: Genus *Mystrium* (Formicidae: Amblyoponinae)

**DOI:** 10.1371/journal.pone.0146170

**Published:** 2016-01-22

**Authors:** Natalie R. Graham, Brian L. Fisher, Derek J. Girman

**Affiliations:** 1 Department of Biology, Sonoma State University, Rohnert Park, California, United States of America; 2 Department of Entomology, California Academy of Sciences, San Francisco, California, United States of America; Field Museum of Natural History, UNITED STATES

## Abstract

The bulk of models used to understand the species diversification on Madagascar have been constructed using vertebrate taxa. It is not clear how these models affect less vagile species that may interact at a variety of spatial scales. Several studies on vertebrates have divided Madagascar into east-west bioclimatic regions, suggesting there is a fundamental division between eastern wet-adapted and western dry-adapted taxa. An alternative model of ecogeographic constraints shows a north-south division. We test whether the diversification in a small arthropod with variable degrees of dispersal conform to either model of ecogeographic constraints proposed for vertebrate taxa. We employ a molecular taxonomic dataset using ~2 kilobases nuDNA (Wg, LW *Rh*, *Abd*-A, 28s) and 790 basepairs mtDNA (CO1), along with geographic and habitat data, to examine the diversification patterns of the ant genus *Mystrium* Roger, 1862, (Subfamily Amblyoponinae) from Madagascar. The nuclear and mitochondrial phylogenies were both congruent with morphospecies as indicated in a recent revision of the genus. Species of *Mystrium* practice different colony reproductive strategies (winged queens vs non-winged queens). Alternate reproductive strategies led to inequalities in female dispersal ability among species, providing an additional layer for examination of the impacts of vagility on divergence, especially when measured using a maternally inherited locus. *Mystrium* species distribution patterns support these models of ecogeographic constraints. Reproductive strategy effected how *Mystrium* mtDNA lineages were associated with large-scale habitat distinctions and various topographical features. Furthermore, in some cases we find microgeographic population structure which appears to have been impacted by localized habitat differences (tsingy limestone formations, littoral forest) on a scale much smaller than that found in vertebrates. The current system offers a finer scale look at species diversification on the island, and helps achieve a more universal understanding of the generation of biodiversity on Madagascar.

## Introduction

Species have evolved in isolation on Madagascar since the breakup of Indo-Madagascar and the northwards drift of India and the Seychelles began 88 million years ago [1]. Today the entire island of Madagascar is a globally recognized biological hotspot, a region of extremely high biodiversity that contains a large number of endemic species [2]. Species radiations on the island have been difficult to adequately explain [3–5] despite the development of diversification models across multiple taxa [6–12]. Although these models have been constructed and evaluated largely using vertebrates, they are being used to understand species diversification and in conservation and management applications for all animal taxa. Many vertebrate species (birds, primates, lizards) typically have relatively high levels of vagility that allow them to disperse across many landscape features. It is not clear how these divergence models effect less vagile species that may interact at a variety of spatial scales [13,14]. Little literature exists for evaluating the species diversification and phylogeographic profiles of taxa with low vagility on Madagascar. Identification of a model taxon group that represents the wide variety of less vagile taxa is critical to evaluating the universality of the proposed models for explaining biodiversity.

Arthropods are being recognized as invaluable for highlighting the influence of fine-scale habitats [14, 15–17]. Ants are a hyperdiverse group of arthropods long utilized as indicators for conservation methods such as surveying and restoration monitoring [17,18]. Surveys and monitoring have typically used a morphospecies approach to distinguish among distinct taxonomic units; however, this approach is time-intensive and therefore limiting [15,19]. Molecular phylogenetic methods within the field of myrmecology have been broadening the ways that ants can be used to gain an understanding of the historical biogeography of a region and in applications for conservation [20,21]. Recent studies have drawn attention to the fine-scale patterns of habitat utilization among ants, demonstrating that this group is particularly well suited to illuminate patterns of endemism on a much smaller scale than vertebrates [5,16,19].

To evaluate the utility of one of these vertebrate derived species diversification models on Madagascar, we used molecular phylogenetic analyses of a genus of ants from Madagascar along with geographic and habitat data to examine the diversification patterns of the ant genus *Mystrium* Roger, 1862, (Subfamily Amblyoponinae). The species in this genus serve as a model for many small arthropods found on Madagascar, as the species examined have been collected in various habitats throughout the island. Furthermore, this group contains species which exhibit two forms of dispersal, colony foundation by either winged (alate) queens or a wingless reproductive caste (ergatoid queens), thus displaying variation in patterns of vagility among females.

### Ecogeographic constraints diversification model

Many of the recent diversification mechanisms proposed for the fauna of Madagascar have been reviewed and the perspectives for testing them concisely outlined [22]. Prominent among these mechanisms is a model of ecogeographic constraints between eastern and western sites or northern and southern sites [6,9]. Madagascar has a north-south orientation, being notably longer than it is wide [23]. The Indian section was attached to the eastern side [24], giving the present island considerable topographic asymmetry [7]. This topography has given rise to fairly discrete habitat types across the island. The primary biomes include an eastern rainforest, western dry-deciduous forest, southern spiny forest, and central grasslands [25,26]. Several studies on vertebrate species [9,12,27,28] have divided Madagascar into east-west bioclimatic regions and much of the northern fourth of Madagascar is included in the eastern rainforest region. Yoder and Heckman (2006) term this model for species differentiation the “ecogeographic constraint.” This hypothesis can be tested by mapping a hierarchical outline of a phylogeny onto localities collected from across the island. Under this ecogeographic model, if there is a fundamental division between eastern wet-adapted and western dry-adapted taxa, we would expect to observe haplotypes from eastern localities and western localities segregating into mutually exclusive clades. Yoder and Heckman (2006) revised their hypothesis of ecogeographic constraints to include the possibility of a north-south split among clades after completing a study of mouse lemurs (genus *Microcebus*). Deep interspecific north-south splits also have been found among Madagascar’s reptiles [27,29]. However, these distinctions are usually associated with large-scale distances among populations separated along the north-south axis of the island rather than by discrete differences in climate.

### *Mystrium* Reproductive Strategies

*Mystrium* species are considered to retain ancestral behavioral and morphological traits [30–33] and are part of a group that contains some of the oldest lineages of ants [34–39]. The workers of the genus *Mystrium* can be distinguished easily from other amblyoponine genera by their characteristic head shape and mandibles; however, in some species, morphological variation within castes in body size and shape, setae or body sculpture is remarkably high, while differences among species can be so slight as to be confounding for taxonomists [40]. A recent morphological revision of the genus *Mystrium* for the Malagasy region including descriptions of six new species was published by Yoshimura and Fisher [40]. Previous taxonomic works [30,41] were insufficient to diagnose the species boundaries of *Mystrium* in the Malagasy region.

One advantage of studying the genus *Mystrium* is that the differences in reproductive strategy among species can provide an additional layer of examination of the impacts of vagility on patterns of divergence within and among species. Different dispersal capabilities among species of *Mystrium*, conferred by alternative reproductive strategies, may be a secondary factor influencing biogeographic patterns of species diversification within the genus [42]. *Mystrium* species exhibit various reproductive forms including typical winged queens, small ergatoid queens [43,44], short-winged queens [45], and intercastes [45,46]. Two major colonial reproductive strategies have been described in ants. During independent colony foundation (ICF), a mated alate queen (winged queen) disperses away from her natal colony and becomes the sole foundress of a new nest, metabolizing her wing muscles to fuel initial brood care, either remaining with the nest (claustral) or leaving the nest untended to forage for food (non-claustral). Alate queens require considerable colony resources while being reared and experience a high mortality rate after leaving the nest [42,47]. Ant species that practice dependent colony foundation (DCF) have a permanently wingless reproductive caste (ergatoid queens) and the colony reproduces by fission or budding. An ergatoid queen that disperses to establish a new nest is accompanied by nestmate workers that provision the initial brood. The small ergatoid queens of *Mystrium* have been described as “multi-purpose” because while they are a reproductive caste they will if left unmated serve in the nurse role played by minor workers in *Mystrium* species that reproduce with winged queens. Smaller ergatoid queens require less per capita energy investment by colonies [47,48]. Therefore there is the possibility that female dispersal will be limited by dependent colony foundation relative to independent colony foundation. If dispersal limitation is the case, it would lead to different patterns of inheritance among maternally inherited loci which would be reflected by patterns of genetic divergence in a mitochondrial phylogeny.

We employed a molecular taxonomic dataset for the elucidation of inter- and intra-species patterns of diversification in the genus *Mystrium*, using ~2 kilobases (kb) of DNA sequence data from four independently segregating nuclear loci and a 790 bp fragment from the cytochrome c oxidase I (CO1) locus adjacent to and overlapping the standard region used in DNA barcoding [49]. We examine *Mystrium* species lineage distribution patterns across the island of Madagascar to determine whether 1) there are discernable differences in biogeographic patterns of female lineages between species with ergatoid versus alate queens, and 2) whether the diversification in a small arthropod with variable degrees of female dispersal conforms to either model of ecogeographic constraints proposed for vertebrate taxa.

## Materials and Methods

### Sampling

Samples in this study are from regions of Madagascar where *Mystrium* was collected during arthropod surveys from 1992 through 2003 [50]. Research, collecting, and export permits were obtained through collaboration with the Ministère de l' Environnement et des Forêts and the Madagascar National Parks. Currently understood species distinctions based on morphological characters were taken into account for sampling (Table 1). *Mystrium oberthueri* Forel (1897), *M*. *voeltzkowi* Forel (1897), *M*. *shadow* Yoshimura and Fisher (2014), *M*. *janovitzi* Yoshimura and Fisher (2014), *M*. *barrybressleri* Yoshimura and Fisher (2014) and *M*. *mirror* Yoshimura and Fisher (2014) are endemic to the island. *M*. *rogeri* Forel (1899) is endemic to Madagascar and Comoros. *Mystrium camillae* Emery (1889) is found across Southeast Asia east to the Philippines and south into Northern Australia. *Mystrium silvestrii* Santschi (1914) is found throughout West and Central Africa. All specimen data for material used in this study are available on AntWeb (www.antweb.org). All voucher specimens are deposited with the California Academy of Sciences, Department of Entomology Collection (CASC). Sequence data has been deposited in Genbank (Table 2). Only samples successfully producing for both primer pairs and considered unique sequences were included in the analyses. For this reason, each sequence often represents haplotypes shared by multiple individuals. Outgroup taxa were chosen based on their relationship to *Mystrium* in higher-level taxonomic studies [34,36,37]. *Adetomyrma* is a recently-described genus endemic to Madagascar [33] which appears to be sister taxa to *Mystrium*. *Stigmatomma* shares some lifestyle characteristics with *Mystrium*; both genera are specialized predators on chilopods, and in some species, queens perform non-destructive cannibalism by cutting holes in the integument of larvae to feed on the exuded hemolymph [51,52].

**Table 1:** List of current *Mystrium* species and species group reproductive types from the most recent revision of the genus by Yoshimura and Fisher (2014).

*camillae* species group			
*barrybressleri*^*a*^	Yoshimura & Fisher, 2014	alate queen	Madagascar
*camillae*^*a*^	Emery, 1889	alate queen	South-East Asia, Australia
*Labyrinth*	Yoshimura & Fisher, 2014	alate queen	Madagascar
*Leonie*	Bihn & Verhaagh, 2007	alate queen	Indonesia
*Maren*	Bihn & Verhaagh, 2007	alate queen	Indonesia
*silvestrii*^*a*^	Santschi, 1914	alate queen	Africa
*mysticum* species group			
*mysticum*	Roger, 1862	alate queen	Madagascar, Comoros
*rogeri*^*a*^	Forel, 1899	alate queen	Madagascar, Comoros
*voeltzkowi* species group			
*eques*	Yoshimura & Fisher, 2014	ergatoid queen	Madagascar
*janovitzi*^*a*^	Yoshimura & Fisher, 2014	ergatoid queen	Madagascar
*mirror*^*a*^	Yoshimura & Fisher, 2014	ergatoid queen	Madagascar
*oberthueri*^*a*^	Forel, 1897	ergatoid queen	Madagascar
*shadow*^*a*^	Yoshimura & Fisher, 2014	ergatoid queen	Madagascar
*voeltzkowi*^*a*^	Forel, 1897	ergatoid queen	Madagascar, Mayotte

^*a*^ Included in current study.

**Table 2:** List of all specimens, museum accession numbers, habitat types, collection locality, and GenBank accession numbers.

Morphospecies	Casent #^a^	Habitat	Locality Code	Lat	Long	CO1	Wg	*Abd*-A	LW *Rh*	28s
*M*. *voeltzkowi*	0500419*	tropical dry forest	Ankarana 210	-12.8636	49.2258	KR063453	KP280018	KR063375	KR063395	KR063415
*M*. *voeltzkowi*	0500393*	tropical dry forest	Sakaramy 325	-12.4689	49.2422	KR063468	KP280016	KR063368	KR063388	KR063408
*M*. *voeltzkowi*	0500425	tropical dry forest	Sakaramy 325	-12.4689	49.2422	KR063479				
*M*. *voeltzkowi*	0500434	tropical dry forest	Sakaramy 325	-12.4689	49.2422	KR063465				
*M*. *voeltzkowi*	0500436	tropical dry forest	Sakaramy 325	-12.4689	49.2422	KR063429				
*M*. *voeltzkowi*	0500394	tropical dry forest	Ankarana 210	-12.8636	49.2258	KR063478				
*M*. *voeltzkowi*	0500437	tropical dry forest	Ankarana 80	-12.9089	49.1097	KR063457				
*M*. *voeltzkowi*	0500435*	Sambirano	Lokobe 30	-13.4194	48.3311	KR063454	KP280019	KR063369	KR063389	KR063409
*M*. *voeltzkowi*	0500397	Sambirano	Lokobe 30	-13.4194	48.3311	KR063480				
*M*. *voeltzkowi*	0500431	tropical dry forest	Ankarana 210	-12.8636	49.2258	KR063481				
*M*. *voeltzkowi*	0500438*	tropical dry forest	Ankarana 80	-12.9089	49.1097	KR063423	KP280025	KR063367	KR063387	KR063407
*M*. *voeltzkowi*	0500392	tropical dry forest	Ankarana 80	-12.9089	49.1097	KR063466				
*M*. *voeltzkowi*	0500429	tropical dry forest	Ankarana 80	-12.9089	49.1097	KR063452				
*M*. *voeltzkowi*	0500415	tropical dry forest	Ankarana 80	-12.9089	49.1097	KR063476				
*M*. *voeltzkowi*	0500424	tropical dry forest	Ankarana 80	-12.9089	49.1097	KR063435				
*M*. *voeltzkowi*_cmplx	0500395	tropical dry forest	Francais 180	-12.3228	49.3381	KR063428				
*M*. *voeltzkowi*_cmplx	0500387	tropical dry forest	Sakaramy 325	-12.4689	49.2422	KR063477				
*M*. *voeltzkowi*_cmplx	0500417*	tropical dry forest	Sakaramy 325	-12.4689	49.2422	KR063470	KP280031	KR063370	KR063390	KR063410
*M*. *mirror*	0500388	tropical dry forest	Anabohazo 120	-14.3089	47.9144	KR063467				
*M*. *mirror*	0500440	tropical dry forest	Anabohazo 120	-14.3089	47.9144	KR063427				
*M*. *mirror*	0501743*	TDF on tsingy	Andranopasazy 150	-18.7094	44.7181	KR063469	KP280030	KR063365	KR063385	KR063405
*M*. *mirror*	0501744	spiny forest	Manantalinjo 150	-24.8169	46.6100	KR063430				
*M*. *mirror*	0501742*	spiny forest	Marie 160	-25.5944	45.1469	KR063475	KP280027	KR063366	KR063386	KR063406
*M*. *mirror*	0500412	tropical dry forest	Anabohazo 120	-14.3089	47.9143	KR063486				
*M*. *shadow*	0500432*	montane rainforest	Ambre 925	-12.5344	49.1794	KR063474	KP280023	KR063371	KR063391	KR063411
*M*. *shadow*	0500439*	rainforest	Ambilanivy 600	-13.7986	48.1617	KR063449	KP280022	KR063374	KR063394	KR063414
*M*. *janovitzi*	0500088*	rainforest	Ankarana 150, 7 km	-12.9000	49.1167		KP280028	KU361295	KU361297	KU361296
*M*. *janovitzi*	0746564	rainforest	Manon 780	-13.9767	48.4233	KU361298				
*M*. *janovitzi*	0746565	rainforest	Manon 400	-13.9617	48.4333	KU361299				
*M*. *oberthueri*	0746569	rainforest	Marojejy 610	-14.4358	49.7606	KR063416				
*M*. *oberthueri*	0746566	lowland rainforest	Amban 25	-15.6833	49.9500	KR063426				
*M*. *oberthueri*	0500105*	lowland rainforest	Amban 25	-15.6833	49.9500	KR063444	KP280020	KR063356	KR063376	KR063396
*M*. *oberthueri*	0746567	rainforest	Amban 425	-15.6667	49.9667	KR063446				
*M*. *oberthueri*	0500091*	rainforest	Amban 425	-15.6667	49.9667	KR063439	KP280021	KR063357	KR063377	KR063397
*M*. *oberthueri*	0500136*	lowland rainforest	Cap Masoala 125	-15.6936	50.1814	KR063434	KP280017	KR063358	KR063378	KR063398
*M*. *oberthueri*	0746568	rainforest	Sandranantitra	-18.0483	49.0917	KR063425				
*M*. *oberthueri*	0500063	rainforest	Sandranantitra	-18.0483	49.0917	KR063455				
*M*. *oberthueri*	0500071*	rainforest	Andriantantely	-18.6950	48.8133	KR063464	KP280029	KR063360	KR063380	KR063400
*M*. *oberthueri*	0746793	rainforest	Andriantantely	-18.6950	48.8133	KR063417				
*M*. *oberthueri*	0500112	rainforest	Amban 25	-15.6813	49.9580	KR063485				
*M*. *oberthueri*	0500138	lowland rainforest	Cap Masoala 125, 1 km	-15.6936	50.1814	KR063484				
*M*. *oberthueri*	0500142	rainforest	Marojejy 610	-14.4358	49.7606	KR063488				
*M*. *rogeri*	0500389	Sambirano	Lokobe 30	-13.4194	48.3311	KR063433				
*M*. *rogeri*	0500391	montane rainforest	Ambre 925	-12.5344	49.1794	KR063459				
*M*. *rogeri*	0500441	montane rainforest	Ambre 925	-12.5344	49.1794	KR063463				
*M*. *rogeri*	0500409*	montane rainforest	Ambre 925	-12.5344	49.1794	KR063461	KP280015	KR063373	KR063393	KR063413
*M*. *rogeri*	0500390*	rainforest	Ambilanivy 600	-13.7986	48.1617	KR063472	KP280013	KR063372	KR063392	KR063412
*M*. *rogeri*	0746571	montane rainforest	Manon 1175	-13.9983	48.4283	KR063460				
*M*. *rogeri*	0500097	montane rainforest	Manon 1175	-13.9983	48.4283	KR063458				
*M*. *rogeri*	0500087	lowland rainforest	Amban 25	-15.6833	49.9500	KR063424				
*M*. *rogeri*	0500102	rainforest	Andri 825	-22.2333	47.0000	KR063443				
*M*. *rogeri*	0746570	rainforest	Andri 785	-22.2167	47.0167	KR063431				
*M*. *rogeri*	0746572	montane rainforest	Ivohibe 8.0 E	-22.4833	46.9683	KR063462				
*M*. *rogeri*	0746573	montane rainforest	Ivohibe 8.0 E	-22.4833	46.9683	KR063471				
*M*. *rogeri*	0500090*	rainforest	Andri 785	-22.2167	47.0167	KR063473	KP280014	KR063361	KR063381	KR063401
*M*. *rogeri*	0746792	montane rainforest	Ivohibe 9.0 NE	-22.4267	46.9383	KR063456				
*M*. *rogeri*	0500119	rainforest	Ando 330	-24.7333	46.8000	KR063432				
*M*. *barrybressleri*	0746560	rainforest	Manon 400	-13.9617	48.4333	KR063441				
*M*. *barrybressleri*	0746559	rainforest	Manon 780	-13.9767	48.4233	KR063440				
*M*. *barrybressleri*	0746558	rainforest	Manon 780	-13.9767	48.4233	KR063442				
*M*. *barrybressleri*	0500082*	rainforest	Anja 875	-14.7500	49.5000	KR063451	KP280026	KR063359	KR063379	KR063399
*M*. *barrybressleri*	0746555	rainforest	Anja 875	-14.7500	49.5000	KR063447				
*M*. *barrybressleri*	0746554	rainforest	Anja 875	-14.7500	49.5000	KR063450				
*M*. *barrybressleri*	0746557	rainforest	Ivohibe 7.5 ENE	-22.4700	46.9600	KR063437				
*M*. *barrybressleri*	0746556	rainforest	Ivohibe 7.5 ENE	-22.4700	46.9600	KR063445				
*M*. *barrybressleri*	0500083	littoral rainforest	Mandena	-24.9517	47.0017	KR063482				
*M*. *barrybressleri*	0746561	littoral rainforest	Mandena	-24.9517	47.0017	KR063438				
*M*. *barrybressleri*	0746562	littoral rainforest	St. Luce	-24.7717	47.1717	KR063436				
*M*. *barrybressleri*	0500114	rainforest	Manon 400	-13.9617	48.4333	KR063487				
*M*. *barrybressleri*	0500079	rainforest	Manon 780	-13.9767	48.4233	KR063489				
*M*. *silvestrii*	0408192	rainforest	Ndakan 360	2.3707	16.1725	KR063421				
*M*. *camillae*	0500094*	K mixed beach forest	Brooketon Coal Mine	5.0100	115.0300	KR063448	KP280024	KR063362	KR063382	KR063402
*M*. *camillae*	0746563	K mixed beach forest	Brooketon Coal Mine	5.0100	115.0300	KR063420				
*Adetomyrma caputleae*	0500384*	montane rainforest	Andranomay 1300	-18.4733	47.9600	KR063483	KP280012	KR063364	KR063384	KR063404
*Stigmatomma mg01*	0500009	rainforest	Ivohibe 7.5 ENE	-22.4700	46.9600	KR063422				
*Stigmatomma mg01*	0500385*	montane rainforest	Ambre 1300	-12.5964	49.1594	KR063418	KP280011	KR063363	KR063383	KR063403
*Stigmatomma tz06*	0500028		Mkomazi	3.9667	37.8000	KR063419				

^a^ Specimens with an asterisk (*) next to the museum accession number are included in the nuclear phylogeny.

### DNA isolation, Amplification, and Sequencing

Genomic DNA was extracted from specimens stored in 95% ethanol at –80°C using Qiagen Dneasy Tissues Kit (Quiagen Inc., Valencia, CA) following the protocol for animal tissues. Individual ants or portions of a specimen were placed in 1.5 ml microtubes and frozen in liquid nitrogen, then ground up thoroughly using a disposable pestle. The material was then digested overnight using 20 uL of 20 mg/mL Proteinase K at 55°C. The lysate was pipetted onto a silica-gel-membrane and purified with a series of ethanol washes using supplied Dneasy Buffers. The DNA was resuspended with 200 uL of 10 mM Tris buffer.

For the mitochondrial phylogeny, two sets of primer pairs from the 5’ end of cytochrome c oxidase I (CO1) gene were chosen from a previous study and used for polymerase chain reaction (PCR) (Table 3). For the nuclear phylogeny, the following loci were chosen from previous studies in which they provided useful resolution for ant phylogenies and were amplified according to the following protocols with some variation in annealing temperature and MgCl_2_ concentration: 398 bp from the wingless locus; 559 bp from the abdominal-A locus; 410 bp from the 28s rRNA locus, and 580 bp from the long-wavelength rhodopsin locus (Table 3). Reactions contained 1.5 mM MgCl_2_, 0.175 mM dNTPs, 0.050 U/ul Taq, 0.540 uM each primer, and 2 uL of template, for a total reaction volume of 10 uL. The amplification protocol consisted of thirty-five cycles of 30 s at 94°C, 1 min at 51°C and 2 min at 72°C, preceded by 3 min at 94°C and followed by a final extension for 10 min at 72°C. The PCR products were purified by exonuclease I and shrimp alkaline phosphatase digestion of single-stranded DNA (primers) and dNTPs (ExoSAP-IT, USB Corporation, Cleveland, Ohio, U.S.A). Samples were sequenced in 10 ul reaction volumes in both forward and reverse directions using the same primers. Dye terminator cycle sequencing was performed using one-eighth the amount of BigDye and the protocol specified by the ABI BigDye Terminator v1.1 Cycle Sequencing Kit on ABI genetic analyzer 3100 (Applied Biosystems, Foster City, CA) at the Core DNA Analysis Facility located at Sonoma State University. Sequences were assembled using the program Sequencher 4.6 (Gene Codes Corporation Inc.). All sequences were confirmed and adjusted by visual inspection of chromatograms. Sequences were relatively straightforward to align, however, LW *Rh* did contain an intron which always occurred at the same location, and was maintained in the dataset during analysis. The concatenated and aligned, four-gene dataset, along with the CO1 dataset, has been deposited with figshare [53].

**Table 3:** Primer sequences for amplification and sequencing of nuclear phylogeny with models of evolution selected by AIC in Modeltest and Mr.Modeltest. Primer sequences used to amplify and sequence the cytochrome oxidase I (CO1) gene for the mitochondrial phylogeny and nuclear mitochondrial-like sequence (NuMts) detection and avoidance.

Locus	Primer	Sequence 5'--->3'	Reference	Model
Wingless	LepWG1F	GARTGYAARTGYCAYGGYATGTCTGG	Brower and DeSalle (1998)	GTR + G
	LepWG2R	ACTICGCRCACCARTGGAATGTRCA	Brower and DeSalle (1998)	
28s rRNA	M06F	CCCCTGAATTTAAGCATAT	Schmitz and Moritz (1994)	HKY + I
	28SCR	CGGTTTCACGTACTCTTGAA	Brady (2003)	
LW *Rh*	LR-143F	GACAAAGTKCCACCRGARATGC	Ward and Downie (2005)	GTR + G
	LR672R	CCRCAMGCVGTCATGTTRCCTTC	Ward and Downie (2005)	
*Abd*-A	AA1172F	CACATCGGCACCGGCGATATGAG	Ward and Downie (2005)	HKY + G
	AA1881R	GGTTGTTGGCAGGATGTCAAAGG	Ward and Downie (2005)	
CO1	M13 CI13F	ATAATTTTTTTTATAGTTATACC	Brady (2003)	
	M13 CI14R	ATTTCTTTTTTTCCTCTTTC	Brady (2003)	
CO1	JerryF	CAACATTTATTTTGATTTTTTGG	Brady (2003)	
	Ben3R	GCWACWACRTAATAKGTATCATG	Brady (2003)	
NuMts	LF1F	ATTCAACCAATCATAAAGATATTGG	Smith *et al*. (2005)	
	TRL-3382R	TYCAWTGCACTTAWTCTGCCATATTA	P.S. Ward personal communication	
	COII-3946R	TATTC ATANCTTCARTATCATTGRTG	P.S. Ward personal communication	

### NuMts Detection and Avoidance

When relatively conserved regions of mtDNA are used to design primers that can amplify mitochondrial fragments from an unknown species, there is a risk of generating paralogous nuclear mitochondrial-like sequences (NuMts). These will cause the number of species to be overestimated if they are treated as orthologous, as a large number of these NuMts have unusually high numbers of point mutations [54]. To eliminate suspicion of pseudogenes we first checked the aligned CO1 data for known diagnostic characters such as stop codons and frameshift mutations by translating the CO1 sequences using the invertebrate genetic code in Bioedit 7.0 [55]. NuMts have been characterized in ants and will show up as an accumulation of base pair differences, generally in the third codon position, at a faster rate than the accumulation of stop codons or indels, so the above-mentioned strategies for detecting these pseudogenes may be ineffective [56]. Consequently, using additional protocols (Ward, pers. comm.), we verified the sequence of fifteen samples with the longest branches and unusually high sequence divergence values. Briefly, after amplifying a longer stretch of mitochondrial DNA with primers LF1 [19] and an ant-customized version of the Pat primer (Ward unpublished) (Table 3), the amplicons were visualized on a TAE gel, cut out, re-amplified with our CO1 primers, and sequenced again under the assumption that most NuMts are less than 1 kb in length [57].

### Analyses

In the mitochondrial phylogeny a total of 72 *Mystrium*, three *Stigmatomma*, and one *Adetomyrma* accessions were included for analysis. The Kimuras two-parameter (K2P) model assumes equal base frequencies and two substitution types and takes into account the fact that transitions and transversions occur at different rates by adding an additional parameter “K,” thereby computing genetic distances while correcting for differences in the frequency of transition/transversion substitutions. We used the K2P model of evolution [58], with 1000 bootstrap replicates for support, to generate a neighbor-joining (NJ) tree [59] using MEGA version 6 [60]. This provided a graphic representation of the among-species divergences, allowing us to group *Mystrium* in MEGA according to morphospecies and calculate mtDNA divergence, both interspecific and intraspecific (within and between group means) also by using the K2P distance model.

For the mitochondrial phylogeny we ran Bayesian analysis and maximum likelihood on Cipress Science Gateway [61]. For Bayesian analysis we first partitioned the data by codon position and then used jModelTest2 [62] to estimate the best nucleotide substitution model for each codon position. For first position and third position the best model determined was GTR+I+ Γ substitution model according to Akaike’s Information Criterion (AIC). The best model determined for the second position was GTR+I. Bayesian inference (BI) analysis was carried out using Mr.Bayes 3.2.4 [63,64]. Starting from random trees, we initiated two individual runs of four Markov-chain Monte Carlo (MCMC) chains, three hot and one “cold,” with ten million iterations each, sampling every 1000 generations. Each run resulted in 10,000 trees and converged on the same topology. The first 25% of samples from the cold chain were conservatively discarded as our “burn-in” percentage. Tracer 2.0 was used to verify that stationarity had been reached (http://beast.bio.ed.ac.uk). A 50% majority-rule consensus tree was generated from the remaining trees and Bayesian posterior probability (pp) values were used as support.

For maximum likelihood analysis we used RAXML [65–67]. First, we partitioned the data by codon position and then set the models to GTR+I+Γ for all positions because of limitations in RAXML. We used the default settings for RAXML and calculated standard bootstrap values based on 1000 replicates, which are used for branch support.

We tested the relationship between geographic distance and genetic distance for *M*. *oberthueri*, *M*. *voeltzkowi*, *M*. *mirror*, *M*. *barrybressleri* and *M*. *rogeri*. First we created a pairwise genetic distance matrix using the K2P distance model in Mega 6.0 for each species [58]. We used GenAlEx 6.501 [68,69] to create pairwise geographic distance matrices and ran a Mantel test for each species using the genetic distances from Mega 6.0. Species that had a significant Mantel test, indicating there was a relationship between genetic distance and geographic distance, were included in a linear regression analysis. We used JMP^®^ Version 11 (SAS Institute Inc., Cary, NC, 1989–2007) to run linear regressions to determine whether the slopes were significantly different among species with alternate colonial reproductive strategies.

In order to verify the species distinctions generated in the mitochondrial phylogeny and to obtain better resolution for deeper nodes in the genus, we also produced a nuclear phylogeny from a concatenated data set which includes a subsample of 19 *Mystrium* and two outgroup accessions and is 1948 basepairs (bp) in length. Samples for this analysis were selected as representatives of each of the species lineages identified in the mitochondrial analysis. Where possible samples representing diverse localities within each species were used to generate sequence data, although not all samples produced sequence across all loci. With a concatenated data set each gene is likely to have different sequence characteristics and rates of evolution. Therefore, we first used jModelTest2 [62] to estimate the substitution model parameters for the four genes individually, then partitioned the data in Mr.Bayes 3.2.4 [63,64], and incorporated the models of gene evolution (Table 3). Bayesian inference (BI) analysis was carried out using the best nucleotide substitution models according to both Akaike’s Information Criterion (AIC) and the likelihood-ratio test (LRT) from jModelTest2. There was no difference between the topology or Bayesian posterior probability (pp) values so only the tree based on AIC-evaluated models is presented.

Bayesian inference (BI) analysis was carried out using Mr.Bayes 3.2.4 [64,64] on CIPRES science gateway [61]. Starting from random trees, we initiated two individual runs of four Markov-chain Monte Carlo (MCMC) chains, three hot and one “cold,” with fifty million iterations each and trees were sampled every 2,000 generations, with an initial seed tree at 12,000 iterations. The analysis did not need to run longer than 50,000,000 generations, because at the end of the run the average standard deviation among topologies was below 0.000001. Each run resulted in 20,000 trees and converged on the same topology. The first 25% of samples from the cold chain were conservatively discarded as “burn-in” percentage. Tracer 2.0 was used to verify that stationarity had been reached (http://beast.bio.ed.ac.uk). A 50% majority-rule consensus tree was generated from the remaining trees. Bayesian posterior probability (pp) values, which represent the percentage of trees sampled after burn-in that recover any particular clade on the tree, were calculated as measures of support.

Maximum likelihood analysis for the nuclear phylogeny was carried out using RAxML [65–67] on the CIPRES science gateway [61]. We partitioned the data by gene and set the models to GTR+I+Γ for all positions because of limitations in RAXML. We used the default settings for RAXML and calculated standard bootstrap values based on 1000 replicates, which are used for branch support. Since RAxML is limited to incorporation of a single model of evolution, we also ran a ML bootstrap analysis using TREEFINDER [70]. We partitioned the data by gene with a data filter, then initiated a ML bootstrap analysis of 1000 replicates and incorporated the models of gene evolution suggested by jModelTest2 [62] and the AIC. A 50% majority-rule consensus tree was generated with bootstrap support. The topologies were consistent between the two methods, with little variation in the support values, and so only the tree produced using RAxML is presented.

## Results

**Fig 1:**
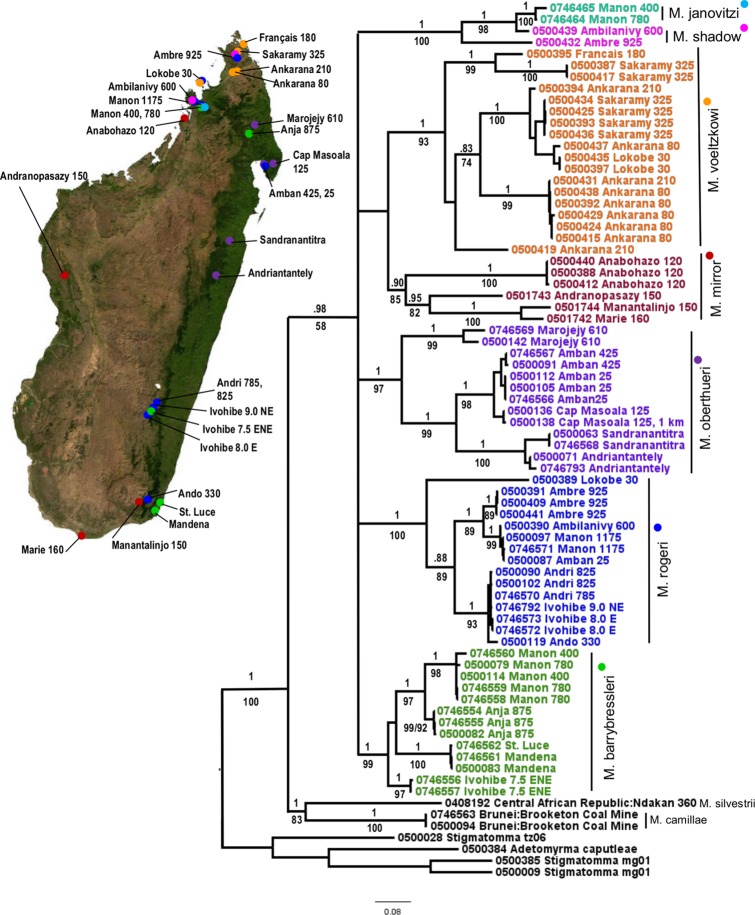
Mitochondrial Phylogeny of *Mystrium*. Maximum likelihood phylogeny based on 790 bp of CO1, summarized as a consensus tree in RaxML. Support values above branches represent Bayesian posterior probabilities (pp), those below branches ML bootstrap. Scale bar shows nucleotide changes per base pair. Taxa are labeled with specimen codes and locality codes. Symbols beside species names on the phylogeny correspond to distribution markers in the adjacent map of Madagascar.

**Fig 2:**
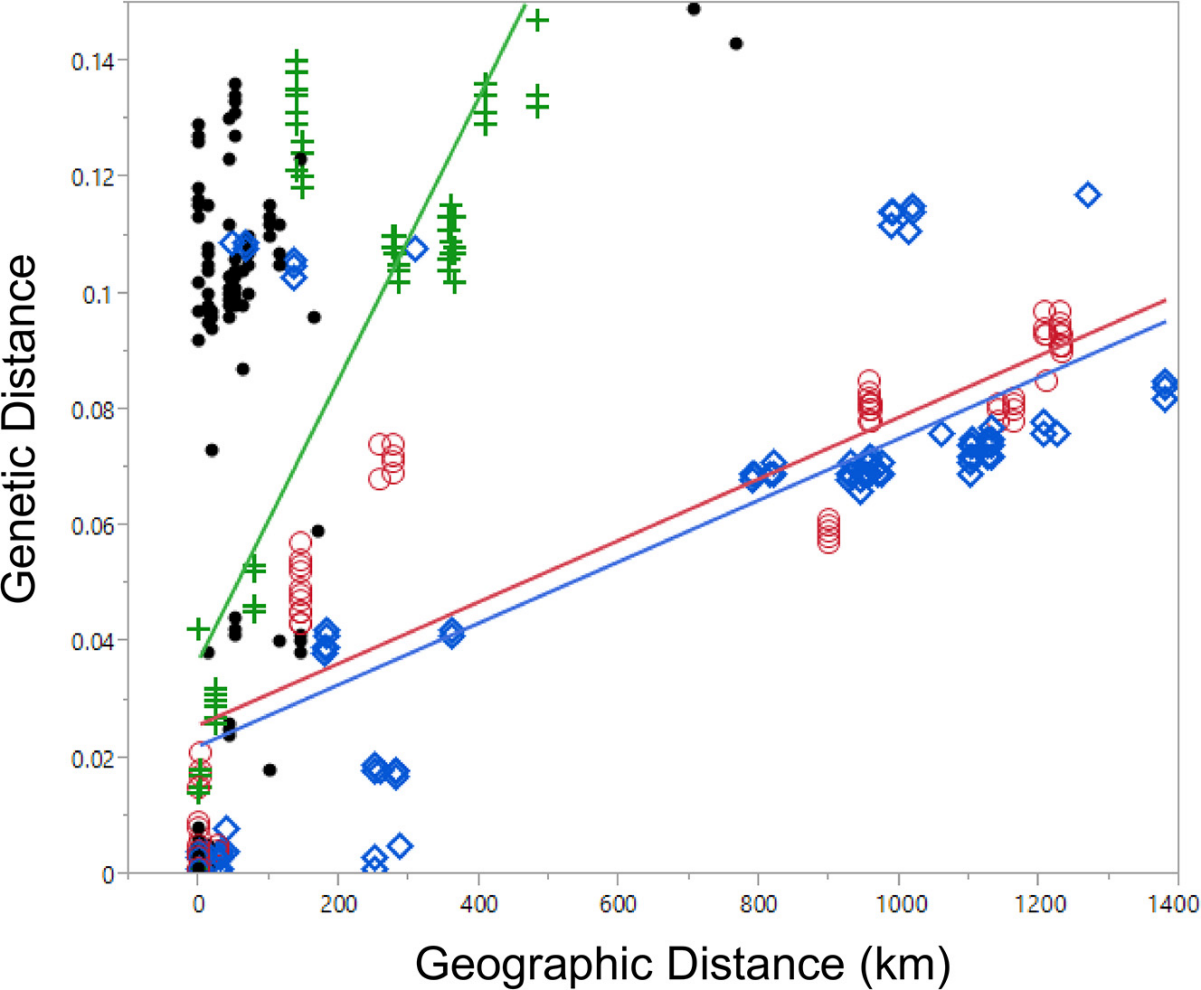
Genetic Distance by Geographic Distance. Linear regression of the relationship of genetic distance to geographic distance by species. The slopes of the lines of the ICF species *M*. *rogeri* (blue diamonds) and *M*. *barrybressleri* (red circles) are not significantly different from each other (F_1_ = 0.000, p = 0.995), but are significantly different from the DCF species *M*. *oberthueri* (green cross) (F_1_ = 111.036, p < .0001). The DCF species *M*. *voeltzkowi* and *M*. *mirror* are represented by black dots and were not included in the regression analysis.

A mitochondrial phylogeny of *Mystrium* species from maximum likelihood analysis, with support from the Bayesian analysis, is presented (Fig 1). Phylogenetic support for published morphospecies and for mtDNA subclades within morphospecies was high across all three methods, despite a few minor differences in topology at distal branches. Average mtDNA congeneric pairwise divergence is 14.28%. Sequences are heavily AT biased; this is normally the case with insect mtDNA [71]. We found no evidence of paralogous nuclear mitochondrial-like genes in the mtDNA data set after eliminating suspect long-branch sequences using long PCR, gel extraction and re-amplification (see Methods). Comparisons of the average K2P divergence values among and within species are listed in Table 4. Average sequence divergence values between *Mystrium* species ranged from 13.44% to 17.67%, whereas average sequence divergence values within *Mystrium* species ranged from 5.23% to 11.69% and are lowest for species which use independent colony foundation. Comparisons of average K2P divergence values within and between subclades of species are listed in Table 5. The slopes of the relationship of genetic distance to geographic distance between ICF and DCF species was significantly different (F_1_ = 111.036, p < .0001). The slopes of the relationship of genetic distance to geographic distance were not significantly different among the ICF species *M*. *barrybressleri* and *M*. *rogeri* (F_1_ = 0.000, p = 0.995) (Fig 2**).**

**Table 4:** species of *Mystrium* calculated using the K2P model of evolution in Mega version 6.0

* *	*M*. *oberthueri*	*M*. *voeltzkowi*	*M*. *rogeri*	*M*. *mirror*	*M*. *barrybressleri*	*M*. *janovitzi*	*M*. *shadow*	*M*. *camillae*	*M*. *silvestrii*
Average within	8.19%	8.16%	5.23%	11.69%	6.92%	1.17%	9.27%	0.13%	
*M*. *oberthueri*									
*M*. *voeltzkowi*	16.01%								
*M*. *rogeri*	15.70%	15.35%							
*M*. *mirror*	16.61%	15.59%	17.10%						
*M*. *barrybressleri*	14.96%	15.40%	14.48%	16.26%					
*M*. *janovitzi*	15.41%	14.70%	13.95%	16.25%	14.26%				
*M*. *shadow*	15.54%	15.93%	15.03%	16.19%	14.06%	6.81%			
*M*. *camillae*	15.86%	16.08%	14.04%	17.67%	13.93%	13.43%	13.44%		
*M*. *silvestrii*	15.96%	15.32%	13.87%	17.61%	13.65%	14.60%	15.96%	12.08%	

**Table 5:** Cytochrome c Oxidase I sequence divergence values within and between subclades of *Mystrium* morphospecies calculated using the K2P model of evolution in Mega version 6.0

*M*. *rogeri*	North	South		
Within subclade	2.47%	0.68%		
North				
South	7.23%			
*M*. *barrybressleri*	Manon	Anja	Ivohibe	Littoral
Within subclade	0.99%	0.34%	0.51%	0.34%
Manon clade				
Anja clade	4.80%			
Ivohibe	8.09%	5.92%		
Mandena/St. Luce	9.27%	7.98%	7.14%	
*M*. *voeltzkowi*	Clade 1	Clade 2	Complex	
Within subclade	0.39%	2.69%	4.86%	
Clade 1				
Clade 2	10.59%			
Complex	12.44%	11.46%		
*M*. *oberthueri*	Marojejy	Pennisula	So. Toam	
Within subclade	4.17%	1.92%	3.52%	
Marojejy				
Pennisula	12.90%			
So. Toamasina	13.63%	10.80%		
*M*. *mirror*	Anabohazo 120	Manatalinjo 150	Marie 160	Andranopasazy150
Within subclade	0.34%			
Anabohazo 120				
Manatalinjo 150	16.01%			
Marie 160	16.84%	5.85%		
Andranopasazy 150	16.52%	14.88%	14.26%	

**Fig 3:**
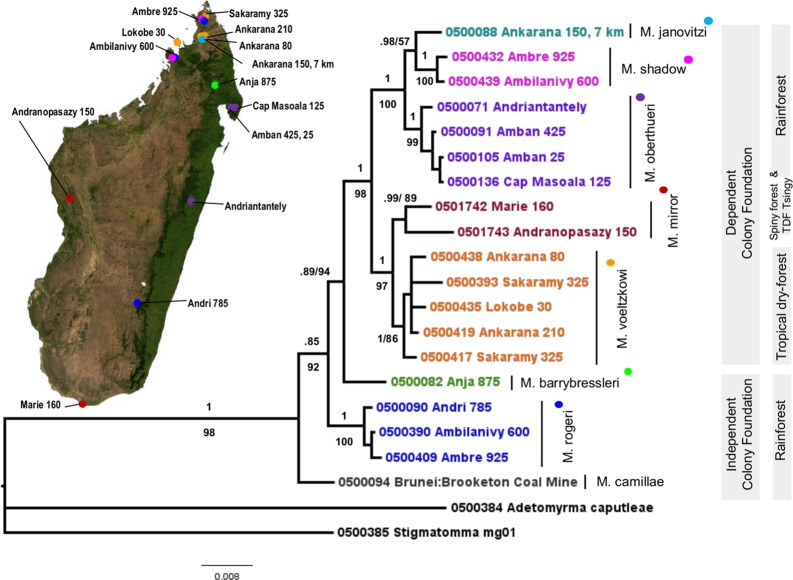
Nuclear Phylogeny of *Mystrium*. Maximum likelihood phylogeny based on 1948 bp of the four nuclear genes Wg, *Abd*-A, LW *Rh*, 28s, summarized as a consensus tree in RaxML. Support values above branches represent Bayesian posterior probabilities (pp), those below branches ML bootstrap. Scale bar shows nucleotide changes per base pair. Colonial reproductive strategy and habitat type are labeled in adjacent bars.

A nuclear phylogeny of *Mystrium* species constructed from the maximum likelihood analysis, with support from Bayesian analysis, is also presented (Fig 3). *Mystrium* is a monophyletic group that is well supported in all analyses, in the mitochondrial phylogeny (1.0 pp, 100% MLbs, 99% NJbs) as well as the nuclear phylogeny (1.0 pp, 98% MLbs). All species of *Mystrium* endemic to Madagascar form their own clade in the mitochondrial phylogeny (.98 pp, 58% MLbs, 72% NJbs) as well as the nuclear phylogeny (.85 pp, 92% MLbs) and the off-island species *M*. *camillae* (nucDNA/mtDNA) and *M*. *silvestrii* (mtDNA only) are basalmost. The species that reproduce by dependent colony foundation, *M*. *shadow*, *M*. *janovitzi*, *M*. *oberthueri*, *M*. *mirror*, and *M*. *voeltzkowi* form a distinct monophyletic group in the nuclear phylogeny (1.0 pp, 98% MLbs).

*Mystrium rogeri* form a well-supported mtDNA clade (1.0 pp, 100% MLbs, 97% NJbs) and a monophyletic group in the nuclear phylogeny (1.0 pp, 100% MLbs). Sequence divergence within *M*. *rogeri* range from a low of 0.127% to a high of 10.759%. In the mtDNA phylogeny, *M*. *rogeri* fall into two clades: a northern clade that is spread across the mountains from the northwest to the northeast encompassing Montagne d’Ambre, R.S. Manongarivo, Foret d’Ambilanivy and down the Masoala peninsula, and a southern clade that is spread across south-central to southern Madagascar (Table 5). An additional mtDNA lineage of *M*. *rogeri* is from the island of Nosy Be (Lokobe 30).

*Mystrium barrybressleri* form a well-supported mtDNA clade (1.0 pp, 99% MLbs, 100% NJbs) and a distinct evolutionary lineage in the nuclear phylogeny. Sequence divergence within *M*. *barrybressleri* range from a low of 0.127% to a high of 9.022%. In the mtDNA phylogeny, *M*. *barrybressleri* contain four subclades restricted by locality: a northwest clade (R.S. Manongarivo), northeast clade (Res. Anjanaharibe-Sud), a south-central clade (R.S. Ivohibe), and a southeast clade (Mandena/St. Luce) (Table 5).

*Mystrium oberthueri* form a well-supported mtDNA clade (1.0 pp, 97% MLbs, 98% NJbs) and a monophyletic group in the nuclear phylogeny (1.0 pp, 99% MLbs). Sequence divergence within *M*. *oberthueri* range from a low of 0.127% to a high of 13.165%. In the mtDNA phylogeny, *M*. *oberthueri* contain three subclades: a Marojejy clade (Marojejy 610), a Masoala peninsula clade (Amban 25, Amban 425, Cap Masoala 125), and a southern Toamasina clade (Andriantantely, Sandranantitra) (Table 5).

*M*. *shadow* and *M*. *janovitzi* form a clade in the mtDNA (1.0 pp, 100% MLbs) and nuclear phylogeny (.98 pp, 57% MLbs). *Mystrium oberthueri* forms a well-supported clade with *M*. *shadow* and *M*. *janovitzi* in the nuclear phylogeny (1.0 pp, 100% MLbs).

*Mystrium mirror* form a well-supported clade in the mtDNA (.90 pp, 85% MLbs, 73% NJbs) and nuclear phylogeny (.99 pp, 89% MLbs). Sequence divergence values within *M*. *mirror* range from a low of 0.0127% to a high of 16.84%. In the mtDNA phylogeny, *M*. *mirror* form one subclade (Forêt d'Anabohazo) and several mtDNA lineages, which were collected at widely dispersed localities, including tropical dry-forest on and off tsingy limestone formations and in spiny desert localities (Table 5). *Mystrium voeltzkowi* and *M*. *mirror* are sister taxa in both the mitochondrial (1.0 pp, 94% MLbs, 76% NJbs) and nuclear phylogenies (1.0 pp, 97% MLbs).

*Mystrium voeltzkowi* form a well-supported mtDNA clade (1.0 pp, 93% MLbs, 93% NJbs) and form a monophyletic group in the nuclear phylogeny (1.0 pp, 86% MLbs). Sequence divergence values of *M*. *voeltzkowi* range from a low of 0.127% to a high of 12.674%. *Mystrium voeltzkowi* was collected in the northern highlands at sites within the Réserve Spéciale de l'Ankarana (Ankarana 210, Ankarana 80), or within the Réserve Spéciale d'Ambre (Sakaramy 325), with two more specimens from the coastal island of Nosy Be (Lokobe 30), and one from Montagne des Français (Français 180, mtDNA phylogeny only). Within the mtDNA phylogeny *M*. *voeltzkowi* form several clades: ‘clade 1’ contains haplotypes collected at Ankarana 80 and Ankarana 210, ‘clade 2’ contains haplotypes collected at Ankarana 80, Ankarana 210, Sakaramy 325, and Lokobe 30 (Table 5). An additional haplotype collected from Ankarana 210 forms an independent mtDNA lineage. A third mtDNA clade (Sakaramy 325 and Français 180) is made up of specimens that were morphologically keyed out to ‘*M*. *voeltzkowi* complex’ by taxonomists at the California Academy of Sciences. Some samples collected within Réserve Spéciale de l'Ankarana (Ankarana 210, Ankarana 80), or within the Réserve Spéciale d'Ambre (Sakaramy 325), have high mtDNA sequence divergence among haplotypes collected at the same site (8.9%-12%) and low divergence among haplotypes collected at sites 71–82 kilometers apart (0.4%-2.5%).

## Discussion

We found unusually high levels of mtDNA sequence divergence within and among the species studied, with subclades within species showing divergence levels greater than typically found among ant species. We found that reproductive strategy appears to have had an effect on how *Mystrium* species female lineages are associated with large-scale habitat distinctions and various topographical features, (i.e. their phylogeography). *Mystrium* species female lineage distribution patterns provide support for both models of ecogeographic constraints [6,9], thus appearing to account for lineage diversification in a manner similar to what was found for vertebrate species. However, in some cases, microgeographic population structure was found to be associated with species which appear to have been impacted by localized habitat differences on a scale much smaller than that found in vertebrates.

### High Levels of Divergence among *Mystrium* Lineages

We recovered strong support in both the nuclear and mitochondrial phylogenies for the species of *Mystrium* included in this study, corresponding with the morphospecies described in the most recent revision of the genus [40]. However, variation between discontinuous evolutionary groupings (subclades) within some morphospecies of *Mystrium* was many times higher than the 2% or 3% mitochondrial sequence divergence threshold that was used to successfully identify the majority of morphospecies of ants in Madagascar [19] and the 1.9% conspecific mitochondrial sequence divergence value measured among ants of North America [19]. Thus, the mtDNA intraspecific sequence divergence values within some lineages of *Mystrium* are values well above those levels typically seen among species of ants. The large amount of intraspecific phenotypic variation found in *Mystrium* may be a reflection of this high genetic divergence. Further work using nuclear loci and expanded sampling will be necessary to confirm the extraordinarily high levels of divergence within species of *Mystrium*.

Within *M*. *mirror* each geographic location consists of a unique clade (Anabohazo 120) or mtDNA lineage (Manatalinjo 150, Marie 160, Andranopasazy 150) and average intraspecific sequence divergence was 11.69%. Intraspecific sequence divergence among the Anabohazo clade and other evolutionary lineages of *M*. *mirror* (16%) rivals sequence divergence levels between *Mystrium* species. Sequence divergence among clades within *M*. *oberthueri* was also high (10.8% to 13.6%). Comparisons between the Marojejy clade and the remaining *M*. *oberthueri* clades yielded the highest intraspecific divergence, likely due to the high mountains and sheer cliffs in the region. These formations are formed from ancient granites and gneisses, materials very resistant to weathering. Our results provide evidence of geographically segregated clades with high phylogenetic divergence within two DCF species of *Mystrium*.

Geographically segregated clades with high sequence divergence in species with ergatoid queens have been found in previous studies with ants [20]. Deep CO1 divergences occur between different collection localities in the ant genera *Odontomachus* and *Anochetus* (*O*. *coquereli*, *A*. *goodmani*, *A*. *boltoni*), which also have ergatoid queens [20,42]. Fisher and Smith (2008) predicted that species with ergatoid queens will have high mtDNA sequence divergence among clades at geographically localized areas because of their reduced dispersal ability. Thus, female-limited dispersal might lead to an extremely site-specific phylogeographic signal in ergatoid queen species.

### Reproductive Strategy Effect on Phylogeography

*Mystrium* species that reproduce by dependent colony foundation form a distinct monophyletic group (Fig 3). The species that reproduce by independent colony foundation, *M*. *barrybressleri* and *M*. *rogeri*, are basal. This suggests that the colony foundation strategy of DCF has arisen once within *Mystrium* and is the derived condition. Dependent colony foundation has been determined to be the derived state in other ants [42] and the condition of the loss of wings is thought to be irreversible [47].

The phylogeographic patterns support the different levels of female dispersal by species that reproduce via independent colony foundation versus species that reproduce via dependent colony foundation. Intraspecific mitochondrial sequence divergence in *M*. *rogeri* and *M*. *barrybressleri* is lower than intraspecific sequence divergence of the Malagasy *Mystrium* reproducing by DCF (Figs 1 and 2). The greater vagility afforded by having winged queens leads to shallower mtDNA divergence within ICF species. *M*. *oberthueri* was used as a proxy for DCF species when we compared the slopes of the lines between ICF and DCF species that showed a pattern of isolation by distance. Within the DCF species *M*. *mirror* divergence values appear to reach a saturation level, making an estimation of the relationship between genetic distance and geographic distance problematic without further sampling (r^2^ = 0.749). We excluded *M*. *voeltzkowi* from the analysis because within *M*. *voeltzkowi* there was also no significant correlation between genetic distance and geographic distance (r^2^ = 0.0259). Instead, geographic barriers to gene flow leading to microendemism in the region may be generating a unique pattern among haplotypes, as discussed below.

We were not surprised to find lower intraspecific divergence values among female lineages in species that reproduce by independent colony foundation because gene flow in ICF species is facilitated by the ability of winged females to traverse greater distances and cross barriers. These same barriers would effectively reduce gene flow in species which have females that reproduce via DCF. In heterogeneous environments consisting of a set of discrete patches that persist for a finite time, movement between patches may only occur by flight, which for females only occurs in species practicing ICF. Conversely, *Mystrium* with ergatoid queens displayed high intraspecific sequence divergence values between conspecifics from different localities. Colonies that practice DCF are limited by terrestrial dispersal from their natal colony and the necessary accompaniment of sister workers. The short-range dispersal of ergatoid queen colonies may have had a severe effect on female gene flow [42, 44, 47, 72, 73]. In a study using mtDNA this low level of gene flow was observed across multiple spatial scales in the population genetic structure of the ant species *Diaccamma cyaneiventre* [72]. High divergence levels are likely a product of minimal genetic exchange among populations. As such, sequence divergence levels within DCF species are expected to be higher.

Although the influence of reproductive strategy appears to impact female dispersal, its impact on each species as a whole remains uncertain because males in these species may be able to disperse more broadly. A rigorous study of within-species variation using nuclear loci would be necessary to elucidate the impact of reproductive strategy on speciation as a whole.

### Large-Scale East-West Ecogeographic Constraints

With a better understanding of the patterns of female dispersal in *Mystrium*, we evaluated a vertebrate-derived biogeographic model of species divergence for ants, which by comparison are likely to have reduced levels of dispersal. Among the largest-scale patterns proposed was that of ecogeographic constriants separating the more mesic rainforest on the eastern side of the island from areas of less mesic conditions of tropical dry forest and spiny desert found on the western side of the island (Figs 1 and 3). The sharp ecological distinction between the humid eastern rainforest and arid western habitat are thought to constitute a barrier to gene flow. Our results suggest that this climatological barrier has caused a basal split between eastern and western clades in the phylogeography of *Mystrium* species. *Mystrium oberthueri* and *M*. *shadow* are both sister taxa and rainforest-restricted species, suggesting that these two species prefer mesic conditions and are hylophilous. The range of *M*. *obertheuri* is on the eastern side of the island and *M*. *shadow* is restricted to rainforest habitat that is part of the eastern climatological rainforest zone which stretches across the northern part of the island. *Mystrium mirror* and *M*. *voeltzkowi* are sister taxa restricted to less mesic habitats, and range from spiny desert in the arid south or tropical dry forest on the western and northwestern side of the island. *Mystrium mirror* has multiple evolutionary lineages that fall out according to habitat (Fig 1). Climate has been correlated with ant diversity, with a combination of temperature and precipitation often representing the best two climatic predictors for diversity of litter-dwelling ants [74]. Thus, east-west phylogenetic splits among species of *Mystrium* support the model for speciation due to ecogeographic constraints proposed for vertebrate taxa [6,9].

### Large-Scale North-South Ecogeographic Constraints

A phylogenetic split was found among haplotypes collected in the north and the south in the ICF species *M*. *barrybressleri* and *M*. *rogeri*. Although it may be an artifact of geographic distance and limited sampling in the center of the island for these species, this split may be related to the island’s mountain ranges. Mountains are thought to have acted as refugia for humid forests during periods of drier climate [75] and offered opportunities for allopatric speciation of populations that remained isolated on mountain tops [10]. The diversification of northern and southern populations of *M*. *rogeri* and *M*. *barrybressleri* during paleoclimatic oscillations may have led to an intraspecific division among haplotypes. The waning and waxing of different climatic regimes during the quaternary is thought to have led to periods when montane vegetation descended a notable distance along the flanks of mountains during warmer and wetter periods or was isolated around summits during cooler and drier periods [76]. The mountains may act as species pumps [27], supplying species that have undergone montane diversification to recolonize surrounding lowland habitat in areas where climatic conditions prevented lowland habitation for a period of geologic time. Having the mountains of the north serve as species pumps for northern lowlands and the mountains of the south serve as species pumps for southern lowlands could explain the deep phylogenetic split between northern and southern haplotypes of *M*. *rogeri* and *M*. *barrybressleri*. Thus, phylogenetic splits among north-south subclades within *Mystrium* potentially support both Yoder and Heckmans (2006) revised model of ecogeographic constraints along the long axis of the island and similar patterns observed in other vertebrate species [28,29]. Further sampling in the intermediate latitudinal range of the island will be essential to test where such a geographic limit between northern and southern clades in the ICF species *M*. *barrybressleri* and *M*. *rogeri* lies, and how intermediate samples will impact the phylogeographic patterns of these species.

### Microendemism: Littoral Forest Habitat

One pattern found did not correspond to established diversification models found in vertebrates: cases of lineage diversification associated with small-scale habitat distinctions. In one of these cases, larger levels of genetic differentiation were found among the southern clades of *M*. *barrybressleri* than among the southern clades of *M*. *rogeri*, both ICF species. One possible explanation for this difference is that some *M*. *barrybressleri* haplotypes were collected from the Mandena/St. Luce locality, which is littoral rainforest habitat (Table 2, Fig 1). Littoral rainforests are narrow patches of coastal forest known to contain unique assemblages of plants and animals [77], and this habitat difference may be a factor in generating an isolated and distinct southeastern Madagascar *M*. *barrybressleri* clade [16]. A similar pattern has been reported previously in other species of Malagasy ants [16] and suggests that the littoral forest habitat may provide a unique habitat harboring microgeographic scales of localized endemism in less vagile organisms.

### Microendemism:Tsingy Habitat

A second pattern of localized patterns of microgeographic distinction among clades occurs in the DCF species *M*. *voeltzkowi* and is associated with the presence of limestone outcrops (tsingy) throughout or surrounding the tropical dry forest in the northern region of Madagascar.

Within *M*. *voeltzkowi*, some haplotypes collected at the same locality show a great deal of mtDNA genetic divergence. The Ankarana tsingy in the northern part of Madagascar appears in patches with a total extension of about 200 km^2^. Here, three *M*. *voeltzkowi* haplotypes from the Réserve Spéciale de l’Ankarana (Ankarana 210) do not group together, and have high (i.e. species level 8.9%–10%) sequence divergence among them (Fig 1). Other *M*. *voeltzkowi* collected together at a different site within the Réserve Spéciale de l'Ankarana (Ankarana 80) also show high sequence divergence values (10.3%). Haplotype sequence divergence is even greater among *M*. *voeltzkowi* collected at the same locality in the Forêt d’Ambre (Sakaramy 325) (12%). In contrast, some *M*. *voeltzkowi* collected at sites 71–82 km apart have relatively low sequence divergence (0.4%–2.5%), as among haplotypes collected at Ankarana 80 and Sakaramy 325 (sequence divergence <1%). Sequence divergence values, therefore, do not relate to the isolation of populations because of distance, but rather the amount of time the populations have been separated, which may have been caused by some other isolating effect such as a physical barrier to gene flow.

Local vicariance events in the tropical dry forest of the north may have caused the long-term isolation of populations of *M*. *voeltzkowi*. Tsingy develops due to direct rainfall on limestone which is very well bedded, clean, has low porosity, and is full of joints [78]. Just as the formation of tsingy is continuous, its destruction is continuous as well; pinnacles (towers of old tsingy) many meters high may fall as side slopes crack or dissolve. Tsingy is an area of constant change, and likely the site of small-scale habitat fragmentation, as patches of deciduous forest exist throughout and around the limestone. For *M*. *voeltzkowi*, changes in the landscape likely have presented effective barriers to gene flow. Segregation of ant populations, time and time again, over the evolution of the Ankarana tsingy is likely to have been the driving factor in the diversification of this species. Likewise, it explains why haplotypes collected farther apart in continuous dry-deciduous forest habitat are more genetically similar than those found closer together in tsingy.

The opposite pattern has been predicted for populations with restricted queen dispersal, especially when measured using maternally inherited mitochondrial genes (mtDNA genes) [79]. Population viscosity is the phenomenon of greater genetic similarity among colonies that are physically nearby in a continuous population than among colonies located further away [79]. This pattern has been observed in species with social insect queens performing DCF that have restricted dispersal, particularly in species without aerial dispersal [42, 72, 73]. Although limited queen dispersal may cause genetic population viscosity, especially when measured using a maternally inherited locus (mtDNA), nuclear genes inherited from both parents may be unaffected, because males can be efficient dispersers and thus mitigate the effects of restricted queen dispersal. Similarly, it would be instructive to include a series of nuclear genes in a broader study to test the patterns of divergence generated here for *M*. *voeltzkowi* and determine if the dispersal of males would degrade the remarkably high divergences among individuals collected at the same localities. However, within the Ankarana tsingy, populations of *M*. *voelzkowi* certainly do not conform to this pattern of population viscosity predicted for colonies of social insects with limited dispersal.

## Conclusions

Here we showed that there were contrasting phylogeographic patterns which occurred in species with differential reproductive abilities when measured using a maternally inherited locus. These patterns are likely a reflection of the influence of colonial reproductive strategy on diversification within the genus *Mystrium*. Using molecular methods we confirmed *Mystrium* morphological species delineation, and demonstrated that the reproductive strategy of dependent colony foundation has likely arisen once. Moreover, we tested one of the prominent proposed mechanisms for vertebrate species diversification with a small colonial invertebrate model and found that similar patterns occurred in species of *Mystrium* ants. However, we also discovered cases of fine-scale micro-endemism in both ICF species (littoral forest clade, *M*. *barrybressleri*) and DCF species (Ankarana tsingy, *M*. *voeltzkowi*). Divergence patterns of *M*. *voeltzkowi*, in particular, suggested that the diversification of female lineages associated with the DCF strategy appear to be especially sensitive to ecological perturbations. In conclusion, this work demonstrates that both testing divergence mechanisms in species with a range of dispersal capabilities and taking into account relevant information regarding species-specific biological mechanisms and life history traits are both invaluable for building a more universal understanding of species diversification mechanisms for regions of high endemism such as Madagascar.
